# ILAE neuroimaging task force highlight: MRI detection of early life epilepsy caused by focal cortical dysplasia

**DOI:** 10.1002/epd2.70038

**Published:** 2025-05-03

**Authors:** Nathan T. Cohen, L. Gilbert Vezina, Chima Oluigbo, Tayyba Anwar, John Archer, Boris C. Bernhardt, Lorenzo Caciagli, Fernando Cendes, Yotin Chinvarun, Luis Concha, Paolo Federico, Eliane Kobayashi, Godwin Ogbole, Stefan Rampp, Anna Elisabetta Vaudano, Z. Irene Wang, Shuang Wang, Gavin P. Winston, William D. Gaillard

**Affiliations:** ^1^ Center for Neuroscience Research Children's National Hospital, the George Washington University School of Medicine Washington DC USA; ^2^ Department of Neurology Austin Health, the University of Melbourne Parkville Melbourne Australia; ^3^ Multimodal Imaging and Connectome Analysis Laboratory McConnell Brain Imaging Centre, Montreal Neurological Institute and Hospital, McGill University Montreal Qubec Canada; ^4^ Department of Neurology, Inselspital, Sleep‐Wake‐Epilepsy‐Center Bern University Hospital, University of Bern Bern Switzerland; ^5^ Department of Neurology University of Campinas – UNICAMP Campinas Brazil; ^6^ Epilepsy Center, Neurology Unit Phramongkutklao Hospital Bangkok Thailand; ^7^ Institute of Neurobiology, Universidad Nacional Autónoma de México Mexico Mexico; ^8^ Hotchkiss Brain Institute Cumming School of Medicine, University of Calgary Calgary Alberta Canada; ^9^ Department of Neurology University of Texas Southwestern Medical Center and Peter O'Donnell Jr. Brain Institute Dallas Texas USA; ^10^ Department of Radiology University of Ibadan Ibadan Nigeria; ^11^ Department of Neurosurgery and Department of Neuroradiology University Hospital Erlangen Erlangen Germany; ^12^ Neurophysiology Unit and Epilepsy Center, AOU Modena Italy; ^13^ Department of Biomedical, Metabolic and Neural Sciences University of Modena and Reggio Emilia Modena Italy; ^14^ Epilepsy Center Neurological Institute, Cleveland Clinic Cleveland Ohio USA; ^15^ Department of Neurology and Epilepsy Center Second Affiliated Hospital, School of Medicine, Zhejiang University Hangzhou China; ^16^ Department of Medicine Queen's University Kingston Ontario Canada

**Keywords:** arterial spin labeling, ASL, drug‐resistant epilepsy, focal cortical dysplasia

## Abstract

The ILAE Neuroimaging Task Force aims to publish educational case reports that highlight basic aspects of neuroimaging in epilepsy consistent with the ILAE's educational mission. Here, we describe a case series of three neonates with focal cortical dysplasia (FCD)‐related drug‐resistant epilepsy who underwent surgical intervention. The purpose of this series is to demonstrate the difficulties of identifying FCD in this age group and to highlight the potential added value of arterial spin labeling MRI for delineation of the epileptogenic zone. This series also supports the importance of the 2019 ILAE recommendations for structural imaging in epilepsy, the 2009 ILAE recommendations on imaging infants and children with recent‐onset epilepsy, and the 2022 ILAE consensus classification of FCD.

**Key points**

- Focal cortical dysplasia (FCD) is a common cause of surgically treatable, drug‐resistant epilepsy in the early life period.
- FCDs are often in electrographic status epilepticus/near status epilepticus.
- Ictal Arterial Spin Labeling (ASL) MRI is a novel imaging modality that can help identify the seizure‐onset zone in early life FCD‐related epilepsy.
- FCDs are distinct on structural MRI before 3 months of age and after 24 months of age; they can be obscured in the interval due to developmental myelination progression.
- T2‐weighted sequences best highlight FCD in the early life period; FLAIR sequences are less helpful.

## 1 INTRODUCTION

The neonatal and infantile period is a time of tremendous change due to neurodevelopmental maturation. There are unique challenges related to the neuroimaging of early‐life epilepsy. Here, we use a case series of three young patients with drug‐resistant focal cortical dysplasia (FCD)‐related epilepsy to describe best practices of neuroimaging in this age group.

## 2 CASES

### 2.1 Case 1

A 2‐month‐old ex‐37‐week female presented with episodic abnormal eye and arm movements noted since her birth. Initially, the frequency of the episodes was one to two times daily, but the day prior to presentation, they increased in frequency up to 10 times, with noted increasing severity. Events were clinically described as forced blinking, followed by left arm dystonic posturing and left facial jerking spreading to involve her left leg. On the day of presentation, the events began to involve her bilateral upper extremities, lasting 10–15 seconds each, with post‐event staring. These were characterized as focal motor seizures with impaired awareness by modified 10–20 neonatal EEG, with interictal FP2/C4 (right frontocentral) spike and polyspike discharges and one to two 30 seconds electroclinical seizures per hour from the same regions. She was initially treated with intravenous lorazepam and fosphenytoin and failed to gain seizure control with intravenous levetiracetam, scheduled clonazepam, topiramate, and oxcarbazepine.

**FIGURE 1:**
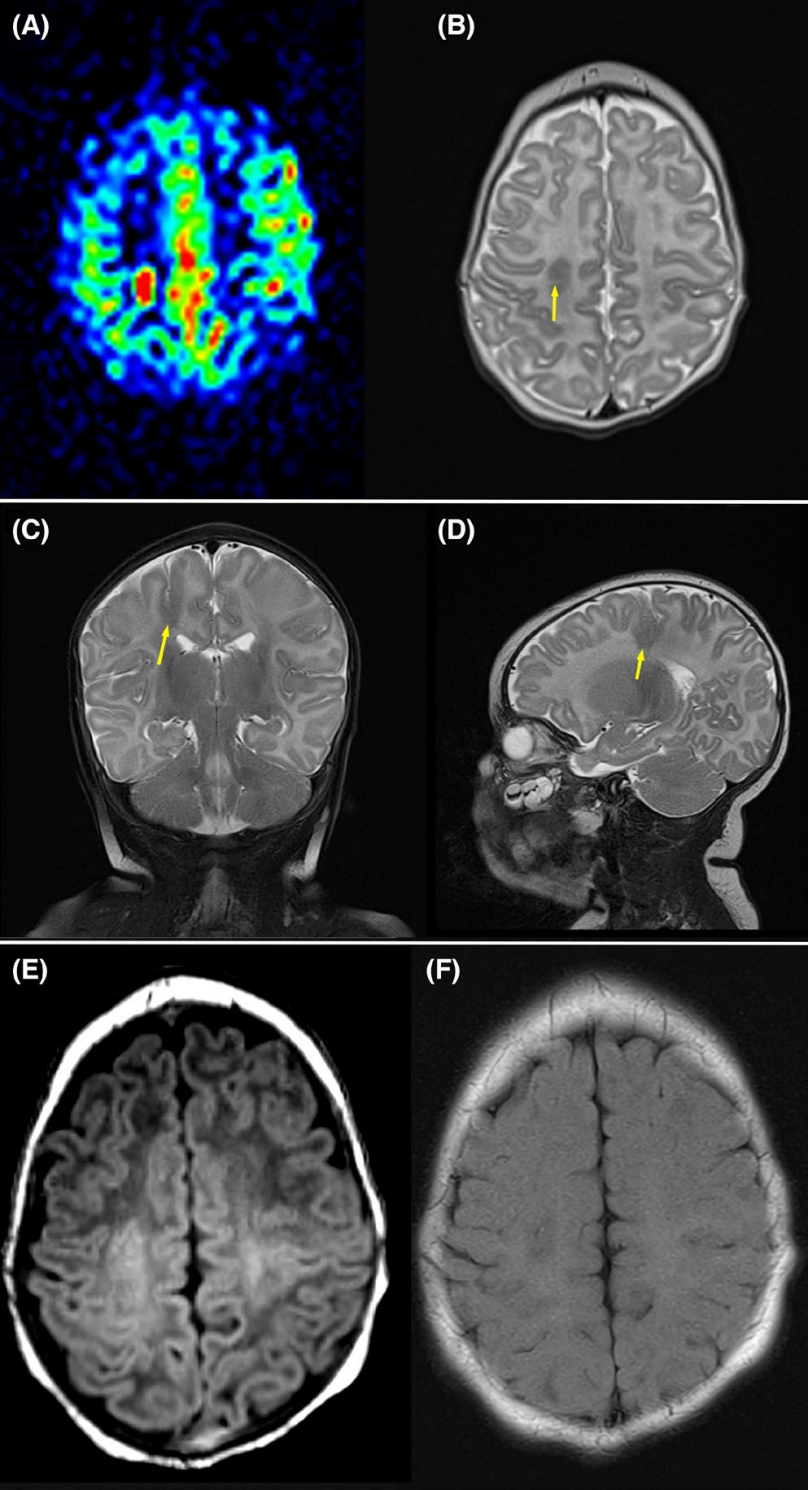
Case 1: Two‐month‐old with right posterior superior frontal bottom‐of‐sulcus FCD IIa. Note that all images in all figures are in radiological orientation. (A) Axial perfusion‐weighted pseudo‐continuous ASL MRI demonstrating hyperperfusion in the region of FCD. No ASL scale available. Slice thickness 4 mm, delay time 1030 ms, pixel size 2.9 mm^2^. (B) Axial T2 with thickening and blurring of gray‐white junction. (C) Coronal T2 with thickening and blurring of gray‐white junction. (D) Sagittal T2 with thickening and blurring of gray‐white junction. (E, F) Note that myelinated subcortical white matter on the left has a similar signal to the right FCD on axial T1 (E), making it less obvious. The FCD is not apparent on axial T2 FLAIR (F), and is most distinct on axial T2 due to better signal contrast (B).

Presurgical 3T MRI brain, acquired on the same day as the EEG, demonstrated cortical thickening and blurring of the gray‐white junction at the posterior inferior margin of the right superior frontal sulcus (Figure 1). This FCD meets the criteria for the described “bottom‐of‐sulcus dysplasia” (BOSD), a described phenotype that is often associated with drug‐resistant epilepsy that is highly amenable to epilepsy surgery (86% seizure‐freedom has been reported).^1^ The axial T2‐weighted image (Figure 1B) shows the inferior margin of the dysplasia with the thickened BOSD corresponding to a region of hyperperfusion as measured with pseudo‐continuous arterial spin labeling (ASL) (Figure 1A). This infantile BOSD is best appreciated on the coronal (Figure 1C) and sagittal (Figure 1D) T2 images with thickening and T2 hypointensity of the inferior aspect of the posterior right superior frontal sulcus. Note that on the axial T1‐weighted (Figure 1E) and axial T2 FLAIR (Figure 1F) images, the dysplasia remains relatively indistinct due to developmentally appropriate immature myelination progression.

Given her age and size, the decision was made to perform an open resection of the MRI‐identified BOSD at the age of 3 months. Postoperative MRI confirmed gross total resection of the right posterior frontal lesion, which was identified pathologically as FCD type IIa. The patient is seizure‐free off antiseizure medications 10.8 months postoperatively.

### 2.2 Case 2

**FIGURE 2:**
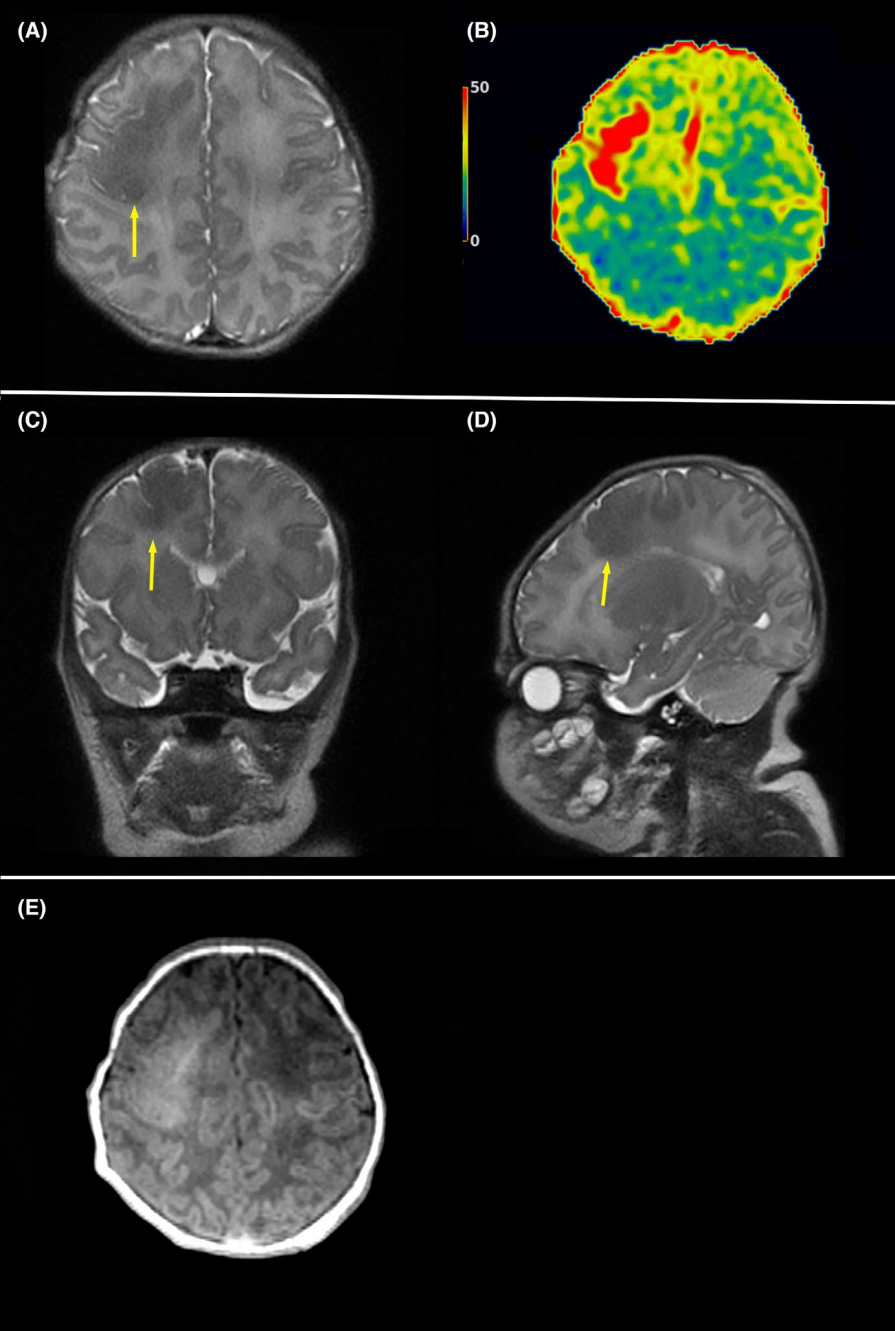
Case 2: One‐day‐old with right frontal FCD IIa. (A) Axial T2 showing right frontal lesion with gyral thickening, blurred gray‐white junction. (B) 3D pseudo‐continuous ASL‐derived cerebral blood flow showing hyperperfusion highlighting the lesion. ASL Scale: mL/100 g/min. slice thickness 3 mm, delay time 1025 msec, pixel size 0.1 mm^2^. (C, D) Coronal and Sagittal T2 showing thickening, blurred gray‐white junction. (E) FCD on T1 axial.

A 1‐day‐old ex‐39 week male was transferred from a birthing hospital for management of status epilepticus. He was placed on continuous video EEG (modified 10–20 neonatal system) monitoring, which captured two seizure types. There were focal motor seizures with myoclonic jerks followed by left upper extremity tonic extension and then left upper extremity and/or left lower extremity clonic movements. The EEG showed semirhythmic spiking at C4 > T4 and Fp2, with subsequent diffuse high amplitude slow wave with overriding low voltage fast activity and further evolution in these channels lasting 30–90 seconds. There were also possible epileptic spasms characterized by abrupt flexion or extension of four extremities with sustained posturing up to 4 seconds. These were characterized by an EEG pattern of diffuse slow wave with overriding fast activity and evolution in the C4/T4 > Fp2 channels to rhythmic theta lasting up to 15 seconds. The status epilepticus was treated with phenobarbital, fosphenytoin, oxcarbazepine, topiramate, and midazolam drip. High‐resolution 3 T epilepsy protocol MRI done at 1 day of life showed a malformation of cortical development in the right posterior superior frontal lobe with cortical thickening and blurring of the gray‐white junction (Figure 2A,C–E). Here, ASL‐derived cerebral blood flow (CBF) demonstrated hyperperfusion in the region of the identified malformation (Figure 2B). The neonate underwent genetic testing with a negative chromosomal microarray. A personalized sequencing panel revealed a heterozygous pathogenic variant in *DEPDC5* (DEPDC5 c.178_181delGAAG; p.Glu60IlefsTer13), a genetic abnormality that is associated with FCD. The child had a subsequent repeat EEG a couple of weeks later, confirming infantile spasms, and was started on vigabatrin. Given ongoing seizures, the child underwent open resection of the right frontal extensive malformation at the age of 6 weeks. The pathology returned as FCD IIa. The child remains seizure‐free and is being maintained on vigabatrin and oxcarbazepine therapy 10.9 months after surgery.

### 2.3 Case 3

**FIGURE 3:**
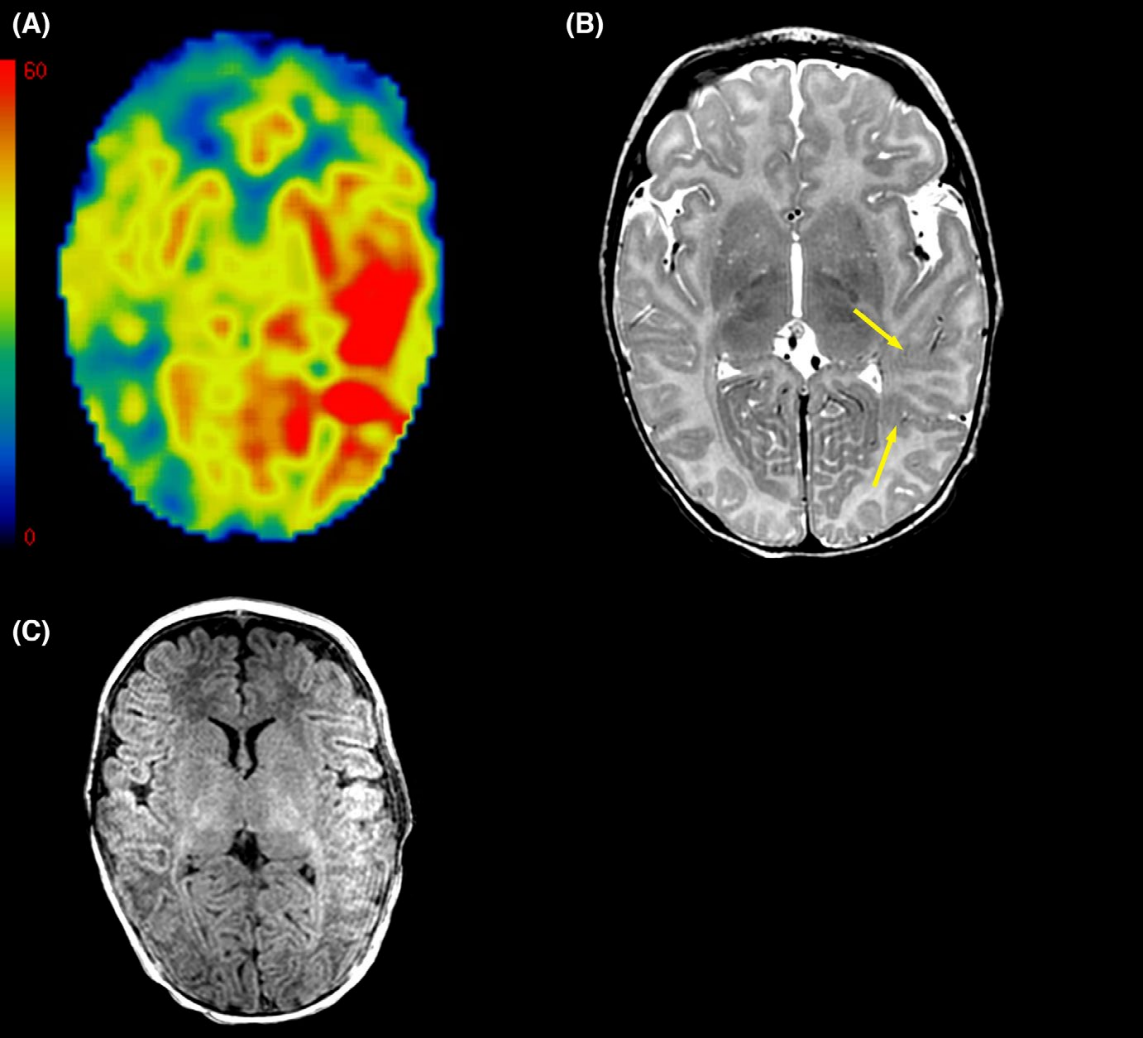
Case 3: Eight‐week‐old with left posterior temporal FCD IIa. Of note, this MRI was initially interpreted as non‐lesional. (A) 3D pseudo‐continuous ASL MRI showing hyperperfusion (bright red) in the region of left posterior temporal FCD. This FCD is much larger than Cases 1 and 2, which is why there is a broader extent of hyperperfusion. Scale is mL/100 g/min. Slice thickness 3 mm, Delay time 1025 ms, pixel size 0.22 mm^2^. (B) Axial T2 showing subtle thickening, poor gray‐white differentiation (yellow arrows), showing the utility of the ASL hyperperfusion in difficult cases. (C) On Axial T1, the lesion is not appreciated.

An 8‐week‐old ex‐41‐week female presented to the neonatal intensive care unit with 2 days of episodes of repetitive blinking, staring, and right‐sided jerking. These continued despite loads of phenobarbital. She was placed on modified 10–20 neonatal EEG, and there were interictal spike‐wave discharges noted at T3/O1, with numerous electroclinical seizures captured that had a signature of monomorphic semirhythmic delta activity at the T3O1 contacts. The initial 3 T epilepsy protocol MRI was interpreted as having no evidence of migrational abnormality. However, re‐review of the initial imaging after a repeat MRI was performed 1 month later due to ongoing worsening seizures revealed a subtle abnormality in the left posterior temporal lobe, with blurring of the cortical medullary interface and an asymmetrically deepened sulcus in the left posterior temporal lobe involving portions of the left middle temporal gyrus and angular gyrus, as well as the posterior junction of the left superior and middle temporal gyri. The ASL MRI demonstrated hyperperfusion in the region of the dysplastic tissue (Figure 3A). Seizures failed to be controlled by phenobarbital, levetiracetam, and oxcarbazepine. The infant eventually developed facilitated infantile spasms in addition to the drug‐resistant focal epilepsy. These were unable to be controlled by vigabatrin therapy.

The infant underwent intraoperative‐MRI‐guided open resection of the left posterior temporal lesion at age 4 months. The pathology was FCD type IIa. The child remains seizure‐free off antiseizure medication for 7.6 years postoperatively.

## 3 DISCUSSION

This case series highlights several key learning points. First, it emphasizes the nebulous imaging characteristics of FCDs and demonstrates the potential augmented detection of subtle FCDs in the neonatal/infantile period with ASL‐MRI hyperperfusion. Second, we note the 2009 ILAE imaging guidelines for infants and children, with particular focus on the special sequences needed in children under 24 months old and the improved yield of T2‐weighted (usually more helpful than T1‐weighted) sequences in this age group and the diminished utility of FLAIR imaging. Third, we review the described BOSD terminology, in which the defined classification has implications for structural/functional imaging findings, genetic underpinnings, and treatment response. Fourth, this series highlights the 2022 ILAE consensus classification on FCD. Finally, we emphasize the importance of epilepsy surgery at the earliest possible time to offer the potential for cure.

This series offers the opportunity to spotlight the differences in ILAE imaging recommendations for young (<24 months old) and older children. Following the recommendations of the ILAE Neuroimaging Task Force consensus on structural imaging in epilepsy, children 2 years and older and adults with new‐onset seizures should have a harmonized neuroimaging of epilepsy structural sequences (HARNESS).^2^ The HARNESS epilepsy MRI protocol includes 3D isotropic T1 at 1 mm^3^ or better, 3D high‐resolution fluid‐attenuated inversion recovery (FLAIR), and two‐dimensional coronal T2 perpendicular to the long axis of the hippocampus. Lesion detection is of the utmost importance as a lesional MRI is associated with 2.5 times higher odds of seizure freedom after surgery.^3^ The 2009 ILAE guidelines for imaging infants, in addition to HARNESS sequences, include T2 sequences in at least two planes. For FCD or other malformations of cortical development, 2 mm thick 2D T2‐weighted images are needed for lesion detection; axial and sagittal planes are preferred. MRIs for children under 24 months old require special attention as immature myelination can affect diagnostic yield: FCDs are more apparent on T2 and may be missed on T1 or FLAIR sequences.^2^, ^4^ The acquisition parameters of T2‐ and T1‐weighted sequences (TR, TE) for infants should be adjusted to optimize the contrast between white and gray matter in MR images. At term, certain structures are initially myelinated (posterior limb internal capsule and corona radiata), and there is orchestrated progression of myelination in a caudocranial (e.g., spinal cord to brain), caudal to rostral, and mesial to lateral sequence; starting in the rolandic cortex to involve splenium of corpus callosum/optic radiations, then occipital/parietal lobes and latest frontal and temporal lobes.^5^ This myelination progression affects MRI interpretation and may affect diagnostic yield: Some FCDs may be initially apparent on MRI, then “disappear” with myelination progression.^6^ Thus, lesions may be initially visible on MRI acquired at <3 months, then become masked by myelination shifts between months 3 and 24, before becoming more conspicuous after 24 months with further progression in myelination. Therefore, in cases where the initial MRI is interpreted as negative, it is recommended to repeat imaging after 24–30 months of age when the myelination pattern is more stable, or sooner if clinically indicated.

Despite its prevalence as the most common etiology of surgically treatable drug‐resistant epilepsy in children and an overall high rate of seizure freedom after surgery,^7^ FCD can be very difficult to detect on MRI.^2^, ^8^ A large, multicenter study of 580 FCD patients found “hotspots” of distribution in the superior frontal sulcus, frontal and temporal poles, and superior temporal gyrus, highlighting these areas as important areas of focus for radiologic review.^9^ In older patients, computer‐aided automated detection techniques have been developed,^10^, ^11^, ^12^, ^13^, ^14^ but these algorithms are not adapted for infants. As in Case 1, the right posterior frontal sulcus BOSD is not clearly visible on the T1 sequence but appears more obvious on the T2 sequence.

ASL‐MRI is a noninvasive perfusion sequence that can quantify CBF without the need for intravenous gadolinium contrast administration. It works based on using radiofrequency pulsations to tag the patient's own arterial blood as a tracer, which can then be used to generate cerebral perfusion maps. Unlike nuclear medicine‐based studies of CBF such as positron emission tomography (H_2_^15^O–PET) or single‐photon emission computed tomography (technetium‐99 m hexamethyl propylenamine oxime (HMPAO) or 99mTC‐ethyl cysteinate dimer (ECD) SPECT), ASL limits exposure to ionizing radiation. Ictal ASL perfusion is an emerging technique that can help to localize the seizure‐onset zone. This is of particular utility in the neonatal/infantile period in which patients with FCD often present in status epilepticus or with acute multiple recurrent seizures. FCDs may be in electrographic status epilepticus/near status epilepticus as confirmed by intracranial stereoelectroencephalography^15^ or other invasive EEG monitoring in which near continuous interictal spiking corresponded to PET hypermetabolism.^16^ There is inter‐vendor variability in ASL quantification; presently, therefore, ASL sequences are reviewed qualitatively and without thresholding. ASL‐detected CBF can remain increased for hours to days after status epilepticus.^17^ This is in contrast to the comparably brief period of ictal hyperperfusion that can be captured from SPECT within 30 s of seizure cessation in older patients, which is then followed by postictal hypoperfusion.^18^ The typical use of interictal ^18^FDG‐PET is to lateralize a seizure focus in a patient with MRI‐negative drug‐resistant epilepsy, including in neonates, by identifying regional hypometabolism.^19^ A study of 498 FDG‐PET scans for pediatric patients with drug‐resistant epilepsy found focal hypermetabolism in 6.6% of cases. This focal hypermetabolism (87% were interictal; 13% ictal) correlated to scalp‐EEG concordant spike count of ≥10 spikes/minute and intracranial EEG evidence of status. Of the 17 patients who underwent focal resection, all had FCD.^16^ Ictal ASL hyperperfusion was first described in neonates in a series of three patients (intraventricular hemorrhage, focal megalencephaly, and hemimegalencephaly), with hyperperfusion described up to 6 days after seizure cessation in one patient.^20^ Ictal ASL hyperperfusion was shown to co‐localize to the electrical source imaging‐defined seizure‐onset zone in a child with FCD and epilepsia partialis continua.^21^ All three cases in the present series had abundant seizures at the time of imaging. The limitations of ASL‐MRI, especially low signal‐to‐noise ratio, among others, are more pronounced in neonates.^22^ Another limitation of ASL (as shown by Case 3) is that the hyperperfusion may extend beyond the obvious MRI‐identified structural abnormality, which in some cases may exaggerate the epileptogenic zone. Further, whereas SPECT measures EEG‐confirmed seizure‐related ictal perfusion, the timing and acquisition of ASL in most pathologies (especially in older patients) may only capture peri‐ or postictal perfusion changes.

The present three cases demonstrate the potential benefit of ASL hyperperfusion in localizing, or confirming, early life FCDs. Ictal CBF studies have comparable utility to interictal FDG‐PET.^23^, ^24^ In real‐world practice, the MRI for Case 3 was deemed non‐lesional, but re‐reviewed demonstrated the subtle findings as highlighted by the associated ASL hyperperfusion. In one study of FCD‐related epilepsy, the authors report that interictal hypoperfusion localizes the dysplasia in about 60% of cases and boosts yield in multimodal imaging up to 90%.^25^ One must be cautious of reliance on hypoperfusion studies, as they may be falsely lateralizing. Interictal H_2_^15^O – PET CBF studies^26^ and interictal single‐photon emission computed tomography (SPECT) can be falsely lateralizing up to 11% of the time in focal epilepsy.^24^ Both ictal and interictal ASL‐MRI require more rigorous evaluation to determine their full clinical utility in FCD and other focal epilepsies. An added layer of complexity is the difficulty of obtaining and analyzing EEG simultaneously with MRI acquisition.

We also emphasize the 2021 neonatal modification of the ILAE classification of seizures and epilepsies.^27^ The majority of neonatal seizures are acute provoked (formerly acute symptomatic) caused by brain insult (e.g., hypoxic–ischemic encephalopathy and vascular (acute perinatal stroke and hemorrhage)) and infection. Epilepsy in this age group is uncommon, and when it occurs, it is most often caused by genetic etiologies, followed by malformations of cortical development. Persistent seizures in neonates should seek to identify genetic and structural etiologies.

The cortical distribution of FCDs in early life epilepsy is understudied. Epilepsy surgical series in the young may be likely biased as surgery is often considered for more severe cases and larger malformations.^28^ There is a recent concept described as multilobar unilateral hypoplasia with severe epilepsy in children that has been used to describe FCD Type Ia cases that present with infantile onset (average seizure onset 0.9 years) drug‐resistant epilepsy, severe cognitive impairment, and nonfocal neurological examination. These cases are often posterior quadrant predominant and have modest surgical outcomes (Engel I in 47% for tailored resections).^29^ However, FCD distribution may actually track more similarly to the “hotspots” as reported in the MELD Atlas.^9^ A large, multicenter, multimodal imaging study by Lee et al. also found preferential distribution of FCD type II to frontal regions with evidence to suggest earlier cessation of neurogenesis and initiation of cellular growth.^30^ A review of the Children's National Hospital FCD database revealed 20 patients with seizure‐onset at less than 120 days of life. Of these 20 patients, FCDs were frontal (*n* = 8, 40%); temporal (*n* = 7, 35%); parietal (*n* = 2, 10%); occipital (*n* = 2, 10%); and multilobar (*n* = 1, 5%). The characterization of early‐life FCD‐related epilepsy deserves further investigation through multicenter studies.

This series reinforces the teaching point of using the multi‐layered classification scheme recommended by the 2022 ILAE Consensus on FCD.^31^ FCDs occur as a spectrum, and to ensure future clinical and research characterization will require inclusion of pathological, radiological, and genetic information, giving as much detail as available into a final summative, integrated diagnosis. For example, in Case 2, the integrated diagnosis would be focal epilepsy with emerging infantile spasms due to right frontal FCD IIa associated with a pathogenic germline *DEPDC5* variant.

Finally, this series underscores the importance of early surgical treatment of FCD. Overall, FCD is amenable to surgical intervention with seizure‐free outcomes achieved in most cases^7^ even with reoperation.^32^ Surgery should be considered, even in those patients with genetic etiologies for the FCD, such as *DEPDC5*, which is associated with high rates of seizure‐free outcomes.^33^ Early surgical intervention is key as long‐term seizure outcomes worsen across time.^9^, ^34^ An international, multicenter study in neonates/infants up to 3‐months‐old (44% with FCD) reported overall ILAE Class I outcomes in 66% of cases.^35^ Of note, surgery was performed during the third month of life in 73% of the cohort. A large, prospective, multicenter study of pediatric epilepsy surgery in children <3 years old compared to those > 3 years old found the former group achieved an Engel I outcome of 59% (and the majority of patients in this group had cortical malformations).^28^ A single‐center retrospective study of epilepsy surgery in early infancy demonstrated seizure freedom or improvement in the majority of cases.^36^ A separate single‐center retrospective study of epilepsy surgery performed before 3 years of age found a 59% Engel I outcome in those children with FCD.^37^ Infantile epileptic spasms syndrome is an epileptic encephalopathy often associated with poor developmental and neurocognitive outcomes. Early surgical intervention can lead to better seizure control and improved developmental outcomes.^38^ In general, there are risks for bleeding for surgery performed at less than 12 weeks of age. Surgery should be performed at ILAE level II pediatric epilepsy centers with skilled teams to achieve the best possible outcomes and to minimize risks.^39^ There is building evidence that FCD pharmacoresistance, clinical characteristics, and surgical outcomes are related to functional network connectivity^40^, ^41^, ^42^, ^43^, ^44^, ^45^ and that early surgical intervention might normalize networks.^46^ Further, newer data suggest that in FCD‐related epilepsy, the vast majority of patients who fail to gain seizure control with one appropriately dosed, appropriately selected antiseizure medication (ASM) will also fail the second ASM. Thus, FCD‐related epilepsy could be considered drug‐resistant after failure of just one ASM, and early surgery could be offered.^7^

In conclusion, this case series of neonatal/infantile FCDs offers several relevant learning points. We highlighted the importance of obtaining infant‐specific modifications for high‐resolution imaging for children under 24 months – T2 sequences in two planes (e.g., axial and sagittal), and the need to review with particular attention to the “hotspots” of FCD including frontal/temporal poles, superior frontal sulci, and superior temporal gyri. We discussed the difficulties associated with the identification of neonatal/infantile FCD, including masking from developmental myelination progression, and showed the augmented value of ictal ASL hyperperfusion in the identification of these dysplasias. Finally, we highlight the importance of early consideration of epilepsy surgery, even during infancy, to provide patients with the chance for seizure freedom.

## CONFLICT OF INTEREST STATEMENT

None of the authors has any conflict of interest to declare.

**Test yourself**

1. The harmonized neuroimaging of epilepsy structural sequences (HARNESS) protocol includes which of the following:

- High‐resolution 3D isotropic T1
- high‐resolution fluid‐attenuated inversion recovery (FLAIR)
- Diffusion tensor imaging
- 2D coronal T2 perpendicular to the long axis of the hippocampi
- Susceptibility weighted imaging

2. The 2022 ILAE Consensus Classification of Focal Cortical Dysplasias recommends a multi‐layered diagnosis that incorporates which of the following features:

- Histopathology diagnosis
- ILAE histopathological subtype
- Genetic findings
- Neuroimaging findings
- Surgical outcomes

3. Neonatal/infantile focal cortical dysplasias:

- May be difficult to appreciate given developmental myelination progression
- May be best appreciated on T2‐weighted sequences
- Always occur as bottom‐of‐sulcus dysplasias
- May have detection augmented by ictal arterial spin labeling MRI hyperperfusion
- Can cause devastating drug‐resistant epilepsy that is amenable to epilepsy surgery

*Answers may be found in the* *Supporting information*

## Supporting information


Data S1.

Data S2.

## Data Availability

The data that support the findings of this study are available on request from the corresponding author. The data are not publicly available due to privacy or ethical restrictions.
