# Supplementary material for: ILAE neuroimaging task force highlight: MRI detection of early life epilepsy caused by focal cortical dysplasia
Source: Epileptic Disord. 2025 May 3;27(4):520–9. doi: 10.1002/epd2.70038 (PMC12353584; doi:10.1002/epd2.70038)
Supplement: Supplementary file 1 — Data S1. [file EPD2-27-520-s001.docx]

Answer

1. a, b, d

2. a, b, c, d

3. a, b, d, e
