# Supplementary material for: ILAE neuroimaging task force highlight: MRI detection of early life epilepsy caused by focal cortical dysplasia
Source: Epileptic Disord. 2025 May 3;27(4):520–9. doi: 10.1002/epd2.70038 (PMC12353584; doi:10.1002/epd2.70038)
Supplement: Supplementary file 2 — Data S2. [file EPD2-27-520-s002.pptx]

## Slide 1
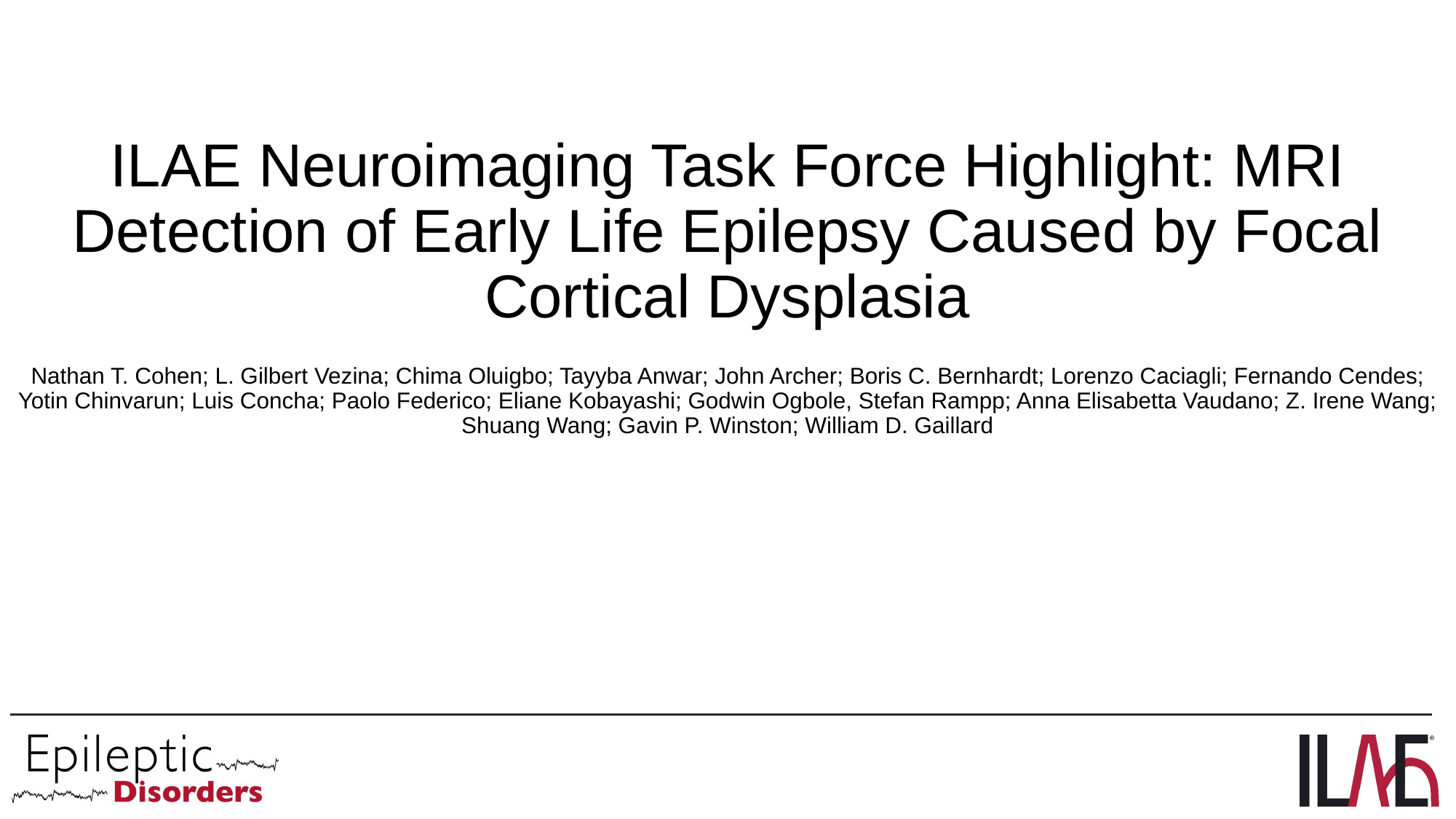

# ILAE Neuroimaging Task Force Highlight: MRI Detection of Early Life Epilepsy Caused by Focal Cortical Dysplasia
Nathan T. Cohen; L. Gilbert Vezina; Chima Oluigbo; Tayyba Anwar; John Archer; Boris C. Bernhardt; Lorenzo Caciagli; Fernando Cendes; Yotin Chinvarun; Luis Concha; Paolo Federico; Eliane Kobayashi; Godwin Ogbole, Stefan Rampp; Anna Elisabetta Vaudano; Z. Irene Wang; Shuang Wang; Gavin P. Winston; William D. Gaillard

## Slide 2
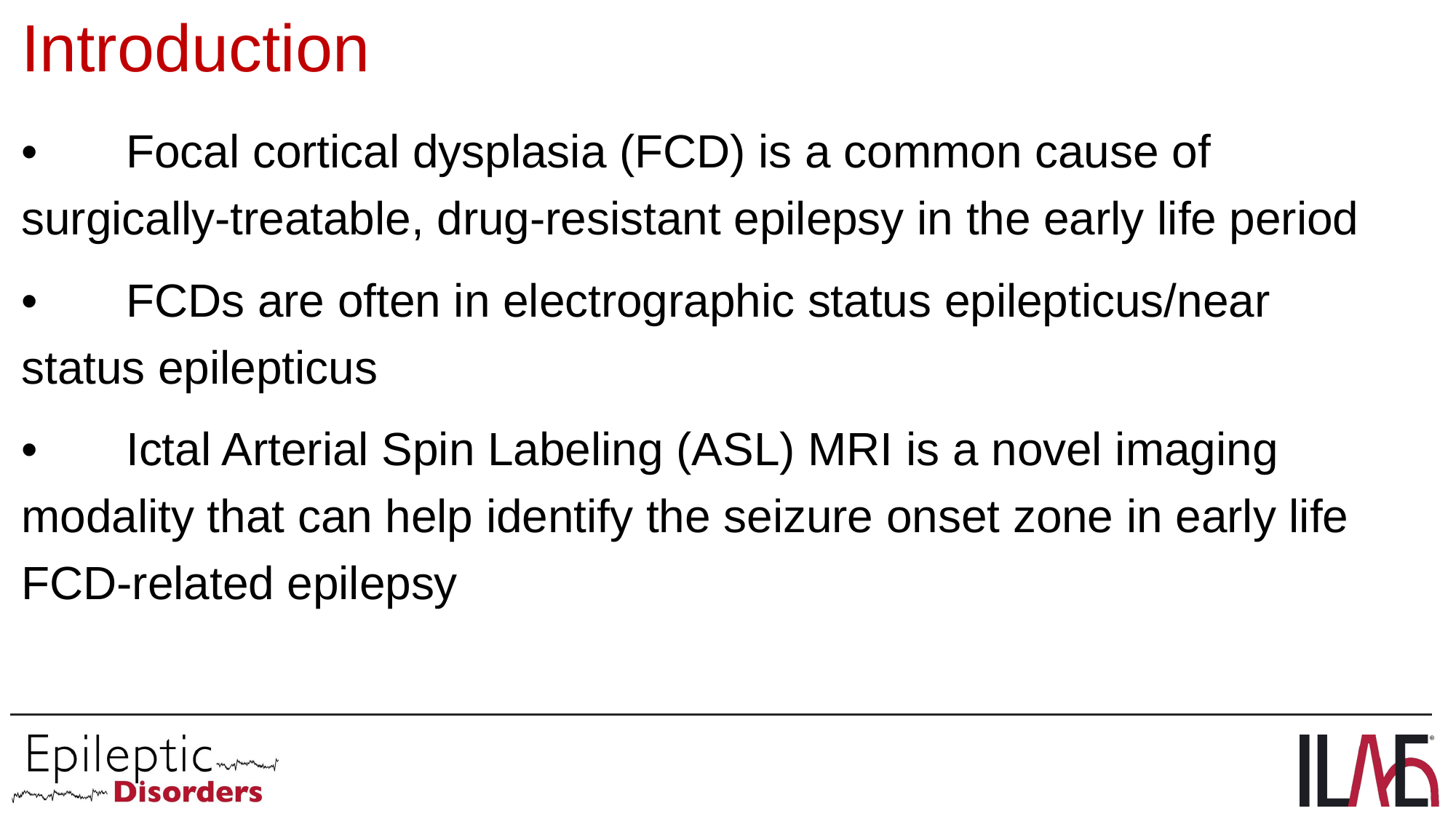

Introduction
•	Focal cortical dysplasia (FCD) is a common cause of surgically-treatable, drug-resistant epilepsy in the early life period
•	FCDs are often in electrographic status epilepticus/near status epilepticus
•	Ictal Arterial Spin Labeling (ASL) MRI is a novel imaging modality that can help identify the seizure onset zone in early life FCD-related epilepsy

## Slide 3
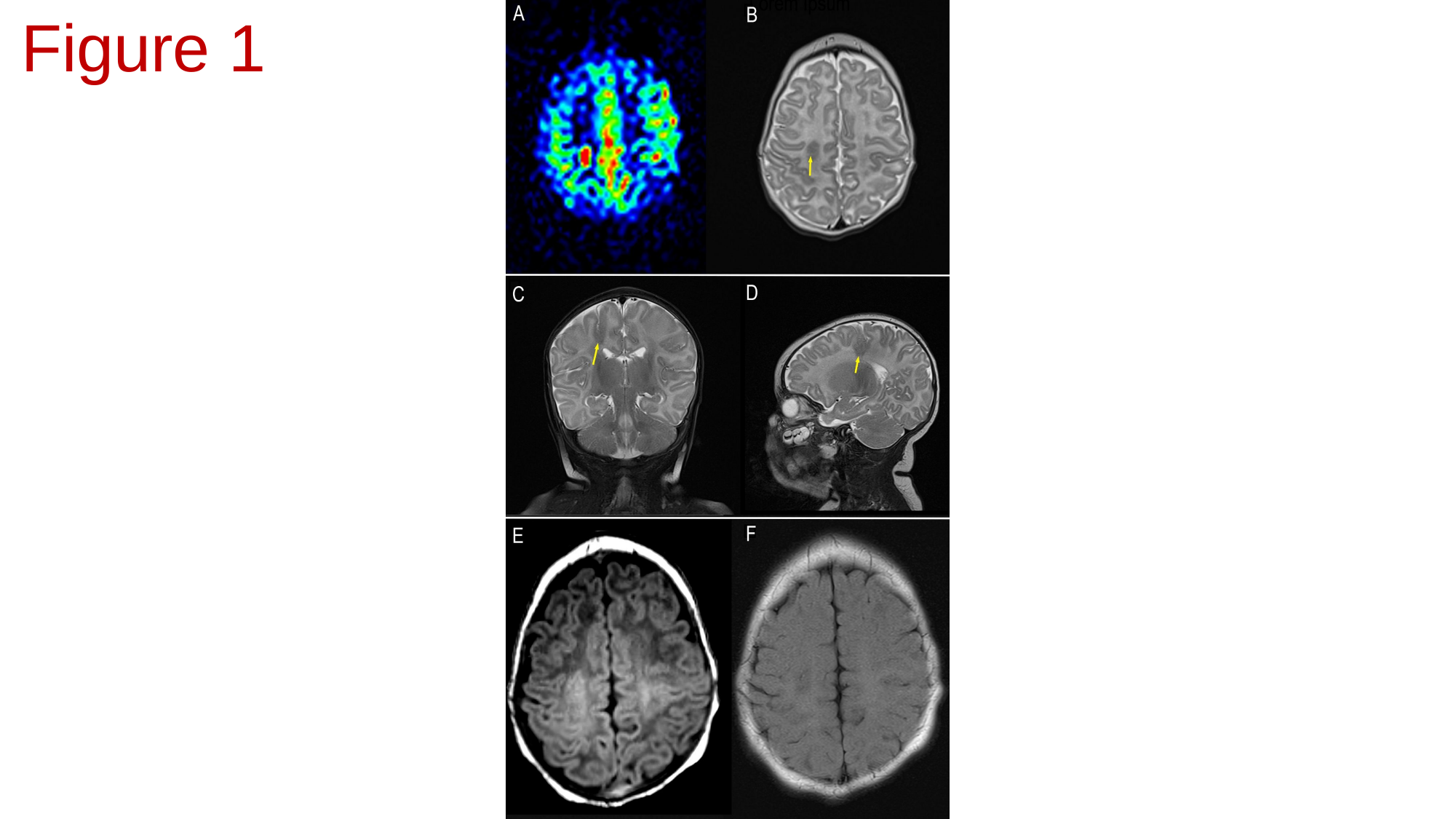

Figure 1

## Slide 4
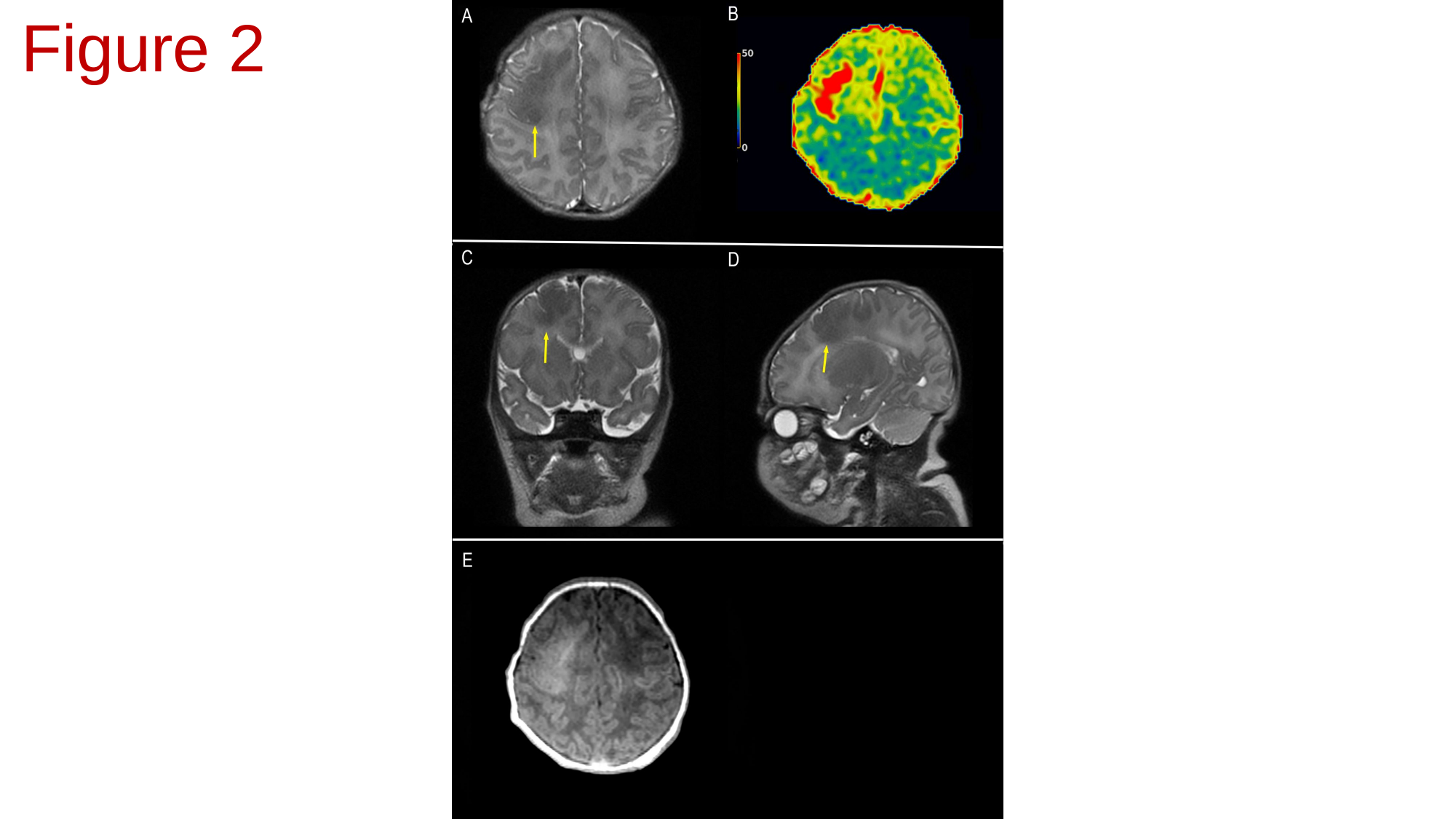

Figure 2

## Slide 5
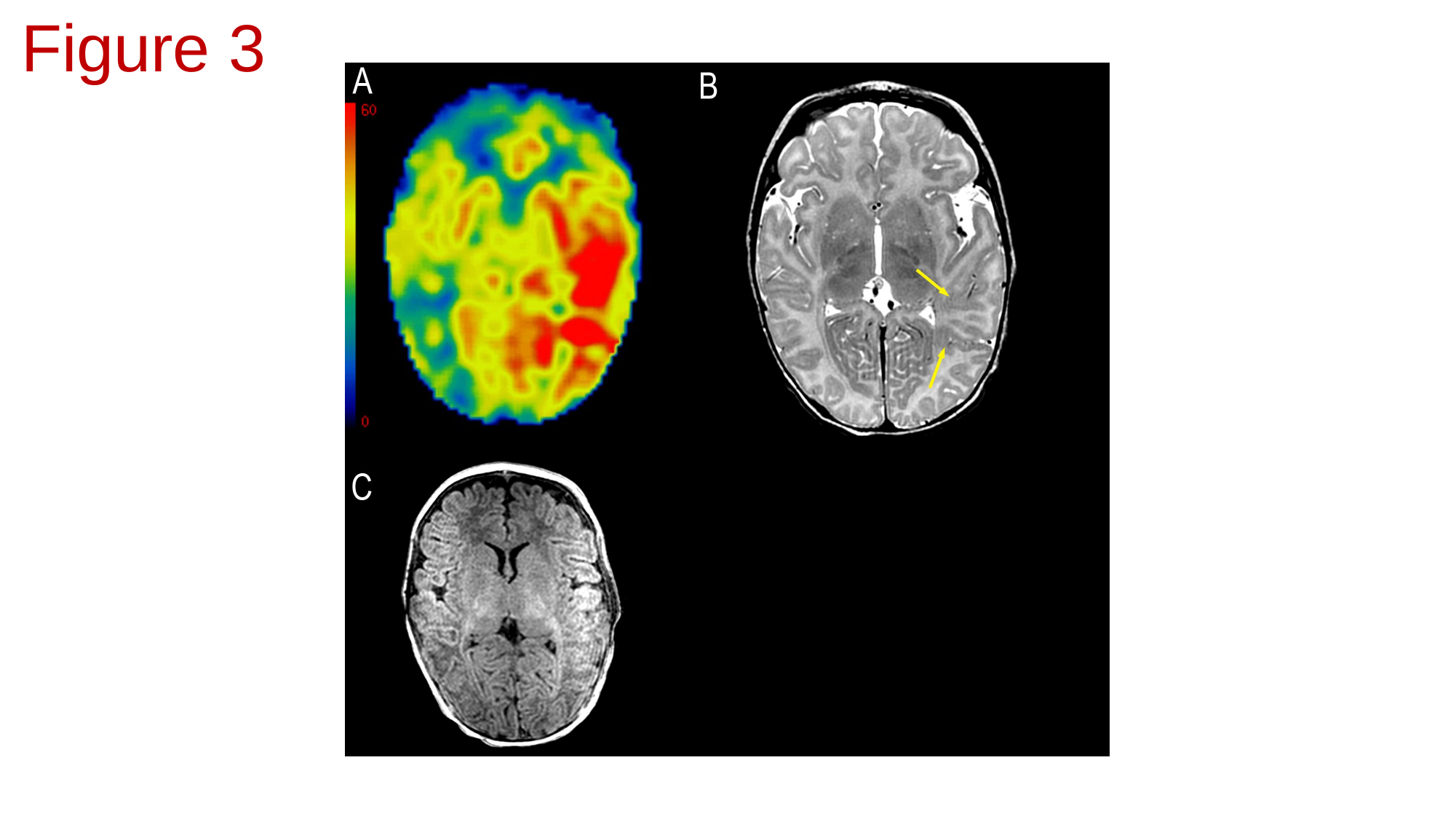

Figure 3

## Slide 6
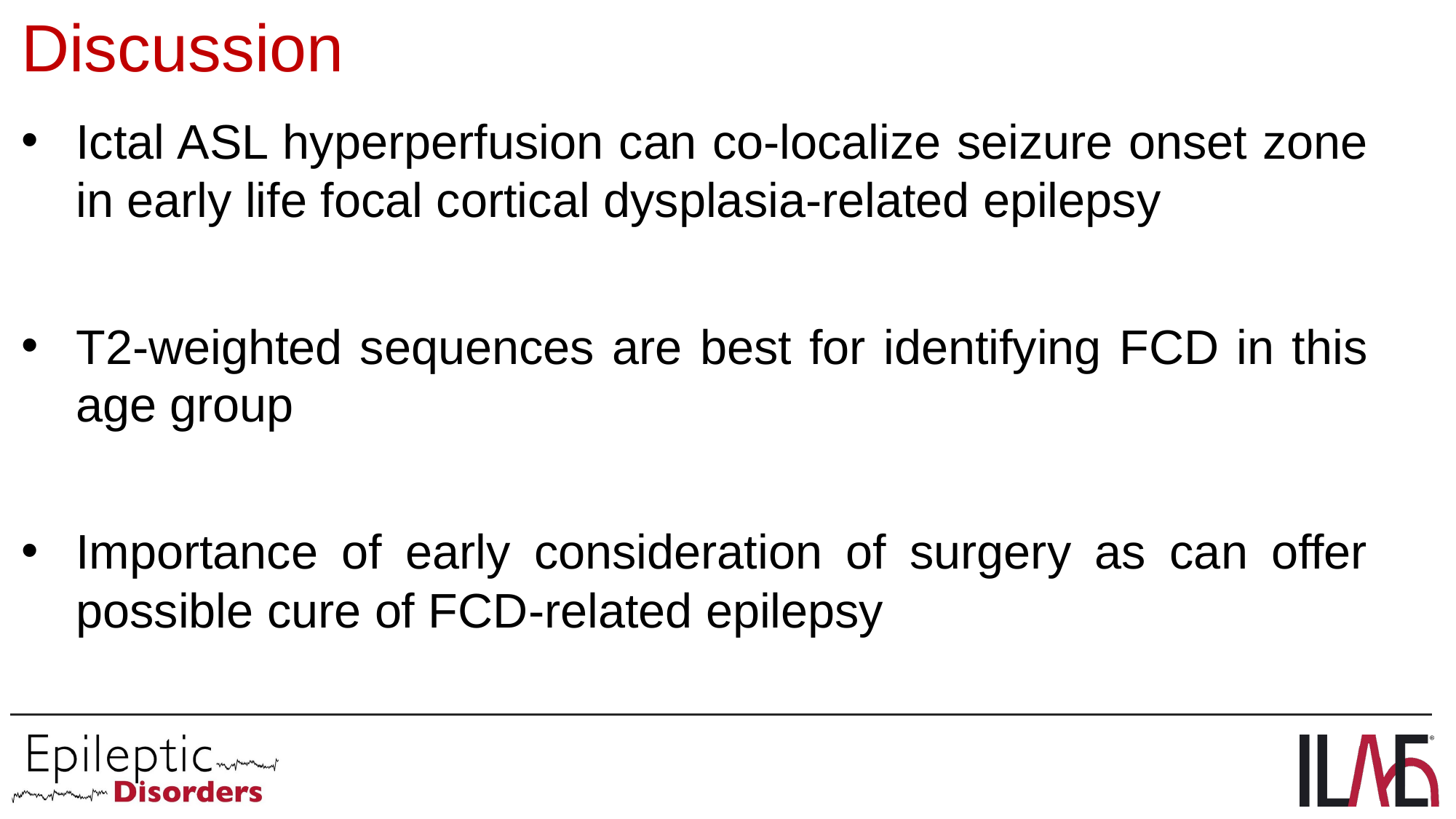

Discussion
Ictal ASL hyperperfusion can co-localize seizure onset zone in early life focal cortical dysplasia-related epilepsy
T2-weighted sequences are best for identifying FCD in this age group
Importance of early consideration of surgery as can offer possible cure of FCD-related epilepsy
